# *Cordyceps militaris* Grown on Germinated *Rhynchosia nulubilis* (GRC) Encapsulated in Chitosan Nanoparticle (GCN) Suppresses Particulate Matter (PM)-Induced Lung Inflammation in Mice

**DOI:** 10.3390/ijms251910642

**Published:** 2024-10-03

**Authors:** Byung-Jin Park, Kyu-Ree Dhong, Hye-Jin Park

**Affiliations:** 1 Department of Food Science and Biotechnology, College of BioNano Technology, Gachon University, Seongnam-si 13120, Republic of Korea; qudwls4733@gmail.com; 2 Magicbullettherapeutics Inc., 150 Yeongdeungpo-ro, Yeongdeungpo-gu, Seoul 07292, Republic of Korea; dhong1233@gmail.com

**Keywords:** *Chitosan nanoparticles*, *C. militaris* grown on germinated *R. nulubilis*, particulate matter anti-inflammatory activity, respiratory disease

## Abstract

*Cordyceps militaris* grown on germinated *Rhynchosia nulubilis* (GRC) exerts various biological effects, including anti-allergic, anti-inflammatory, and immune-regulatory effects. In this study, we investigated the anti-inflammatory effects of GRC encapsulated in chitosan nanoparticles (CN) against particulate matter (PM)-induced lung inflammation. Optimal CN (CN6) (CHI: TPP *w/w* ratio of 4:1; TPP pH 2) exhibited a zeta potential of +22.77 mV, suitable for GRC encapsulation. At different GRC concentrations, higher levels (60 and 120 mg/mL) led to increased negative zeta potential, enhancing stability. The optimal GRC concentration for maximum entrapment (31.4 ± 1.35%) and loading efficiency (7.6 ± 0.33%) of GRC encapsulated in CN (GCN) was 8 mg/mL with a diameter of 146.1 ± 54 nm and zeta potential of +30.68. In vivo studies revealed that administering 300 mg/kg of GCN significantly decreased the infiltration of macrophages and T cells in the lung tissues of PM-treated mice, as shown by immunohistochemical analysis of CD4 and F4/80 markers. Additionally, GCN ameliorated PM-induced lung tissue damage, inflammatory cell infiltration, and alveolar septal hypertrophy. GCN also decreased total cells and neutrophils, showing notable anti-inflammatory effects in the bronchoalveolar lavage fluid (BALF) from PM-exposed mice, compared to GRC. Next the anti-inflammatory properties of GCN were further explored in PM- and LPS-exposed RAW264.7 cells; it significantly reduced PM- and LPS-induced cell death, NO production, and levels of inflammatory cytokine mRNAs (IL-1β, IL-6, and COX-2). GCN also suppressed NF-κB/MAPK signaling pathways by reducing levels of p-NF-κB, p-ERK, and p-c-Jun proteins, indicating its potential in managing PM-related inflammatory lung disease. Furthermore, GCN significantly reduced PM- and LPS-induced ROS production. The enhanced bioavailability of GRC components was demonstrated by an increase in fluorescence intensity in the intestinal absorption study using FITC-GCN. Our data indicated that GCN exhibited enhanced bioavailability and potent anti-inflammatory and antioxidant effects in cells and in vivo, making it a promising candidate for mitigating PM-induced lung inflammation and oxidative stress.

## 1. Introduction

Particulate matter (PM) is one of the major environmental factors for exacerbating respiratory, skin, and cardiopulmonary diseases in China, Korea, and Japan, with the effects varying by particle size [1,2,3]. PM (particles > 10 µm in diameter) is effectively filtered out by the cilia and mucus in the nasal passages and upper respiratory tract [4]. PM with a diameter 10 μm or less, known as PM_10_, can reach deep into the respiratory system due to its small size [5]. PM_10_ enters the respiratory system through the nose and mouth, and due to its small size, it can reach deeper parts of the respiratory tract, such as the trachea, bronchus, alveoli, and even lung parenchyma. Furthermore, as the particle diameter decreases, its ability to penetrate deeper into the respiratory system increases. For instance, PM_2.5_ (particles 2.5 μm or less in diameter) can reach the bronchioles and alveoli, leading to more severe health impacts. These fine particles pose significant risks to both the pulmonary and cardiovascular systems, making them particularly harmful [6,7,8,9,10]. Moreover, ultrafine particles (PM_0.1_), with diameters of 0.1 µm or less, pose an even greater risk due to their ability to efficiently pass through the respiratory tract and the alveolar–capillary barrier, entering the bloodstream. This allows PM_0.1_ to be distributed throughout the body, potentially causing systemic effects and contributing to diseases beyond the lungs, such as cardiovascular and neurological conditions [11].

PM can worsen respiratory illnesses in individuals with preexisting lung conditions such as chronic obstructive pulmonary disease (COPD) and cystic fibrosis due to various factors such as biological components, organic compounds, and inorganic compounds [12]. Biological components such as LPS from *Legionella pneumophila*, *Pseudomonas aeruginosa*, *Haemophilus influenzae*, and *Aeromonas* species, can induce inflammation and lung tissue damage by activating the TLR4 pathway and exaggerating the innate immune response [13]. In addition, PM can damage bronchial epithelial cells which could increase bacterial infection as well as colonization [14,15,16,17,18]. This process enhances bacterial adhesion, triggers inflammation, and exacerbates COPD by further damaging lung tissues [19]. Additionally, transition metals such as aluminum, iron, copper, chromium, nickel, and manganese, along with organic compounds like quinones and polycyclic aromatic hydrocarbons (PAHs), present in PM generate reactive oxygen species (ROS). These ROS, including superoxide radicals and hydroxyl radicals, further contribute to oxidative stress and inflammation through various chemical reactions, exacerbating respiratory diseases [20,21,22]. Numerous studies have demonstrated a strong correlation between exposure to PM and the onset of respiratory conditions such as asthma and lung cancer. The detrimental impacts of PM are linked to several mechanisms including inducing oxidative stress, weakening epithelial barrier function, and amplifying pro-inflammatory signaling cascades [1,23,24]. To mitigate the detrimental impact of PM on respiratory health, bioactive compounds from natural sources offer a promising strategy. Previous studies have demonstrated the efficacy of various bioactive compounds derived from *Cordyceps militaris* grown on germinated *Rhynchosia nulubilis* (GRC) [25,26,27,28,29,30,31]. Compounds such as cordycepin, proteins, soyasaponins, isoflavones, and polysaccharides are widely used to treat a number of diseases and conditions, including inflammation and immune-related disorders, cancer, high blood sugar, hyperlipidemia, respiratory and heart disease [25,26,27,28,29,30,31]. These compounds also increase the levels and activity of antioxidant enzymes such as catalase (CAT), superoxide dismutase (SOD), and glutathione peroxidase, and inhibit the expression of inflammatory cytokines [31,32,33,34,35,36].

However, many active compounds in the extract of GRC become inactive or inert when orally administered. For example, cordycepin and adenosine, the main bioactive compounds in GRC, are rapidly metabolized by adenosine deaminase, resulting in the production of inactive metabolites [37]. Phenolic compounds, including isoflavones and pro-anthocyanidins, exhibit poor bioavailability as they are poorly absorbed, rapidly metabolized, and eliminated from the body, which limits their biological and pharmacological effects [38,39].

To resolve this issue, nanotechnology is used for encapsulating active compounds in derived from natural product extracts and treating various diseases, including inflammatory diseases, cancer, and chronic human diseases. This approach enhances the bioavailability, targeting capabilities, and controlled-release profiles of the natural products [40,41,42]. Therefore, we applied a nanomedicine drug delivery system to effectively transport bioactive compounds from GRC to target tissues, thereby improving their bioavailability.

Nanoparticle sizes, ranging from 10 to 1000 nm, affect their cellular uptake, tissue penetration, and targeting capacities [43]. They also improve the solubility of water-soluble compounds, increase the half-life of systemic circulation by reducing immunogenicity, sustain the release of active compounds in the target organ, and lower the frequency of administration [44,45,46,47]. However, serious concerns regarding carbon- and metal-based nanoparticles have been raised owing to their toxicological impact on humans [48,49]. After entering the human body, they accumulate in the heart, liver, spleen, kidneys, and brain, causing mitochondrial damage, DNA alterations, and even cell death [48]. Among nanoparticles, chitosan (CHI) nanoparticles (CNs) draw attention as drug delivery carriers because they exhibit low toxicity through biodegradability, versatile routes of administration biocompatibility, and mucoadhesive activity compared to carbon-based and metal-based materials [48,49]. This natural polymer is digested by enzymes such as lysozyme and lipase present in serum or the digestive system, which are non-toxic [40]. Therefore, CNs are extensively utilized in the fields of nanomedicine for the delivery of hydrophobic compounds, including drugs, vitamins, proteins, nutrients, and phenolic compounds [40]. CNs are used to enhance the drug delivery of grape, *Elsholtzia splendens*, and *Mentha longifolia* extracts with natural medicinal properties [38,50,51]. However, the encapsulation of GRC extract in CNs has not yet been investigated.

Therefore, in this study, we synthesized GRC encapsulated in CNs (GCN) and evaluated the size and zeta potential of nanoparticles and their physicochemical properties to develop an optimal delivery system. Subsequently, the anti-inflammatory and reactive oxygen species (ROS) scavenging effects of GCN were verified in the RAW264.7 cells and murine model exposed to PM_2.5_. Additionally, the enhanced bioavailability of GCN in mouse small intestinal tissue was investigated to confirm its potential as a novel nanomedicine for the treatment of inflammatory lung diseases.

## 2. Results

### 2.1. GCN Synthesis

**Figure 1:**
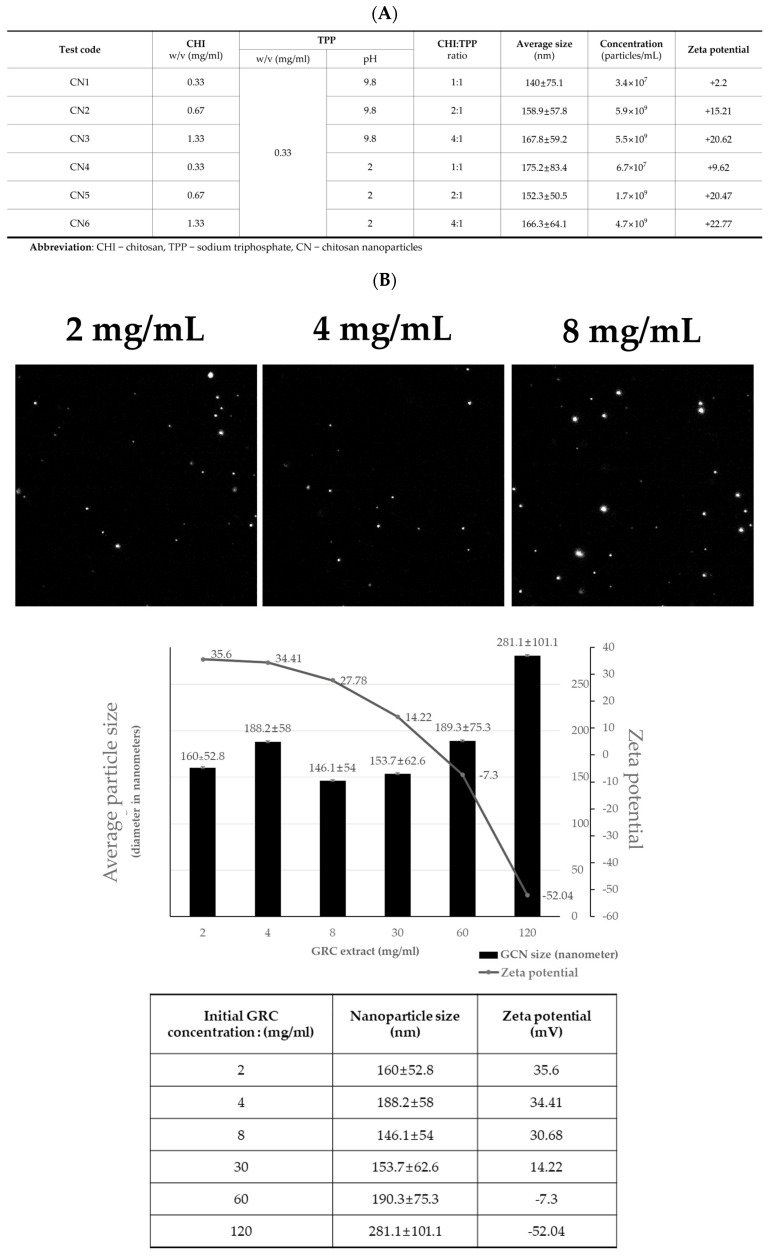
Optimal GRC encapsulated in chitosan nanoparticles (GCN) synthesis. (**A**) Particle sizes, concentrations, and zeta potentials of chitosan (CHI) nanoparticles (CN) with different CHI ratios at pH 9.8 and 2.and (**B**) Representative NTA images of GCN (2, 4, and 8 mg/mL) and particle size distribution and zeta potential results of GCN at various GRC concentrations (2, 4, 8, 30, 60, and 120 mg/mL).

CNs were synthesized using ionic crosslinking between tripolyphosphate (TPP) and CHI. The size and zeta potential varied with the CHI:TPP ratio and the TPP pH value (Figure 1A) [52,53]. Despite these variations, there was no significant difference in the average nanoparticle size when TPP was at pH 2 compared to pH 9. However, as the pH decreased, the zeta potential increased slightly, indicating that more amino groups (NH_3_^+^) were exposed on the CN surface due to the decreased negative charge of TPP reducing its reactivity with CHI. The optimal condition, CN6 (CHI: TPP *w/w* ratio of 4:1; TPP pH 2), exhibited the zeta potential of +22.77 mV, ideal for GRC encapsulation (Figure 1B).

At various GRC concentrations (2, 4, 8, 30, 60, and 120 mg/mL), the GRC chitosan nanoparticles (GCN) showed increased negative zeta potential at higher concentrations (60 and 120 mg/mL). At a concentration of 8 mg/mL, the encapsulation of GRC extract under CN conditions (CHI: TPP *w/w* ratio of 4:1; TPP pH 2) increased the zeta potential from +22.77 mV to +30.68 mV. This increase in zeta potential improved stability by minimizing particle agglomeration [54].

### 2.2. CN Attenuates Inflammatory Cell Infiltration in PM-Treated Lung Tissue

**Figure 2:**
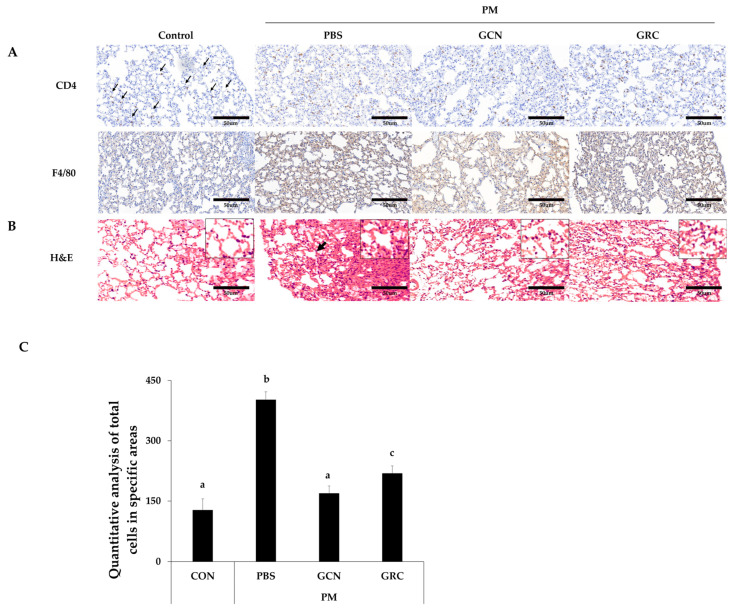
GCN mitigates macrophage and T-helper-cells infiltration and reduces inflammatory gene expression in lung tissue of PM-stimulated mice. (**A**) Representative images of CD4 and F4/80 immunohistochemical staining (scale bar: 50 µm, magnification: 200×). and (**B**) lung tissue of control, PM, PM + GCN and PM + GRC-treated mice stained with hematoxylin and eosin (H&E) (scale bar: 50 µm, 200× magnification) and (**C**) the total number of cells in the lung tissue section. The experiments were repeated three times (*n* > 3) and results are presented as mean ± SD. ^a–c^ Bars with different letters differ significantly at *p* < 0.05 by Tukey HSD test. PM (particulate matter), GCN (GRC encapsulated in chitosan nanoparticles). Control = mouse treated with PBS; negative control group = mouse treated with particulate matter and PBS; GCN = mouse treated with particulate matter and (GCN 300 mg/kg/day); GRC = mouse treated with particulate matter and GRC (300 mg/kg/day).

To explore the inhibitory effects of GCN on macrophage and T-cell infiltration in the lung tissues of PM-treated mice, immunohistochemical analyses were conducted using CD4 and F4/80 as markers for helper T cells and macrophages, respectively. As depicted in Figure 2A, administration of PM alone led to a significant increase in the infiltration of F4/80-positive macrophages and CD4-positive helper T cells within lung sections. However, this infiltration was notably reduced following treatment with GCN (300 mg/kg) and GRC (300 mg/kg), achieving statistical significance, and GCN led to a more significant reduction in total cell numbers than observed in the PM-exposed group (*p* < 0.05).

Furthermore, histological assessments of lung tissue from PM-exposed mice, as shown in Figure 2B, revealed that while the control group’s lung tissues maintained normal architecture without pathological alterations, PM exposure resulted in significant pathological changes. These included inflammatory cell infiltration and alveolar septal hypertrophy [55]. Treatment with GRC (300 mg/kg) and, more significantly, with GCN (300 mg/kg) ameliorated these pathological changes (Figure 2B).

### 2.3. GCN Alleviated PM-Induced Inflammatory Cell Infiltration in BALF

**Figure 3:**
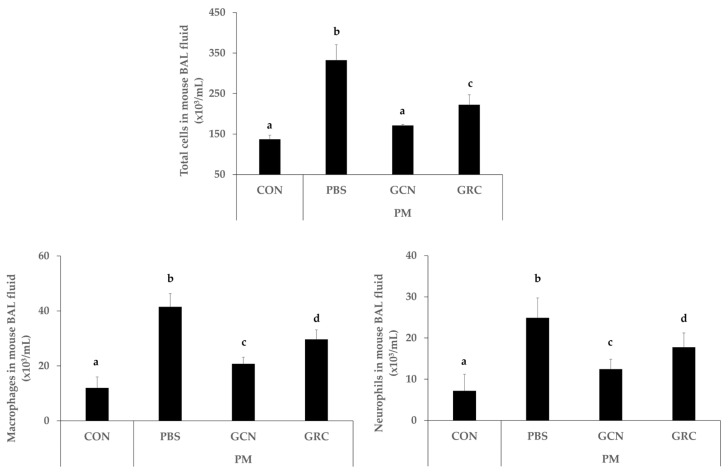
GCN reduced the number of infiltrated total cells in bronchoalveolar lavage fluid (BALF) of mice with PM-induced airway inflammation. Total number of cells in BALF of 6-week PM-exposed mice by group. The experiments were repeated three times (*n* > 3) and results are presented as mean ± SD. ^a–d^ Bars with different letters differ significantly at *p* < 0.05 by Tukey HSD test. PM (particulate matter), GCN (GRC encapsulated in chitosan nanoparticles). Control = mouse treated with PBS; negative control group = mouse treated with particulate matter and PBS; GCN = mouse treated with particulate matter and (GCN 300 mg/kg/day); GRC = mouse treated with particulate matter and GRC (300 mg/kg/day).

To elucidate the effect of GCN on inflammatory cell infiltration in the bronchioles and alveoli in the lungs, we measured the number of neutrophils and total cells in the bronchoalveolar lavage fluid (BALF) (Figure 3).

The results demonstrate a significant increase in the total cells (Figure 3) in the BALF of mice treated with PM compared to the control group (*p* < 0.05).

Treatment with 300 mg/kg of either GCN or GRC notably decreased the numbers of both total cells and neutrophils in the BALF compared to the PM group (*p* < 0.05). Notably, GCN led to a more significant reduction in total cell numbers than was observed in the PM-exposed group (*p* < 0.05).

### 2.4. GCN Attenuates Cytokine Expression in PM-Treated Lung Tissue

**Figure 4:**
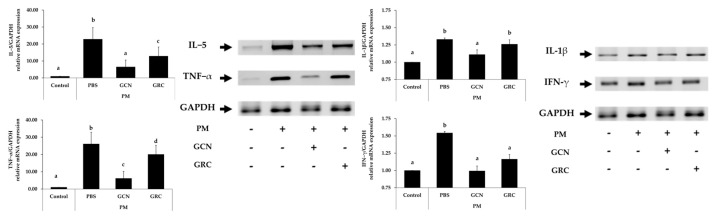
GCN reduces inflammatory gene expression in lung tissue of PM-exposed mice. The level of TNF−α, IL−5, IL−1β, and INF-γ mRNAs expression were measured. The experiments were repeated three times (*n* > 3) and results are presented as mean ± SD. ^a−d^ Bars with different letters differ significantly at *p* < 0.05 by Tukey HSD test. PM (particulate matter), GCN (GRC encapsulated in chitosan nanoparticles). Control = mouse treated with PBS; negative control group = mouse treated with particulate matter and PBS; GCN = mouse treated with particulate matter and GCN (300 mg/kg/day); GRC = mouse treated with particulate matter and GRC (300 mg/kg/day).

To evaluate the anti-inflammatory effects of GCN in mice exposed to PM, we examined whether the level of inflammatory cytokine mRNA expression was reduced in lung tissue. PM exposure elevated the level of inflammatory cytokine mRNAs expression in mouse lung tissues. We evaluated the inhibitory effect of GCN on the levels of TNF-α, IL-5, IL-1β, and INF-γ mRNA expression. As illustrated in Figure 4, PM exposure significantly increased these cytokine mRNA levels compared to the control group (*p* < 0.05). GCN markedly reduced PM-induced TNF-α and IL-5 mRNA expression, compared to GRC (*p* < 0.05).

### 2.5. GCN Inhibits the Expression of Pro-Inflammatory Cytokines in PM-Stimulated RAW264.7 Cells

**Figure 5:**
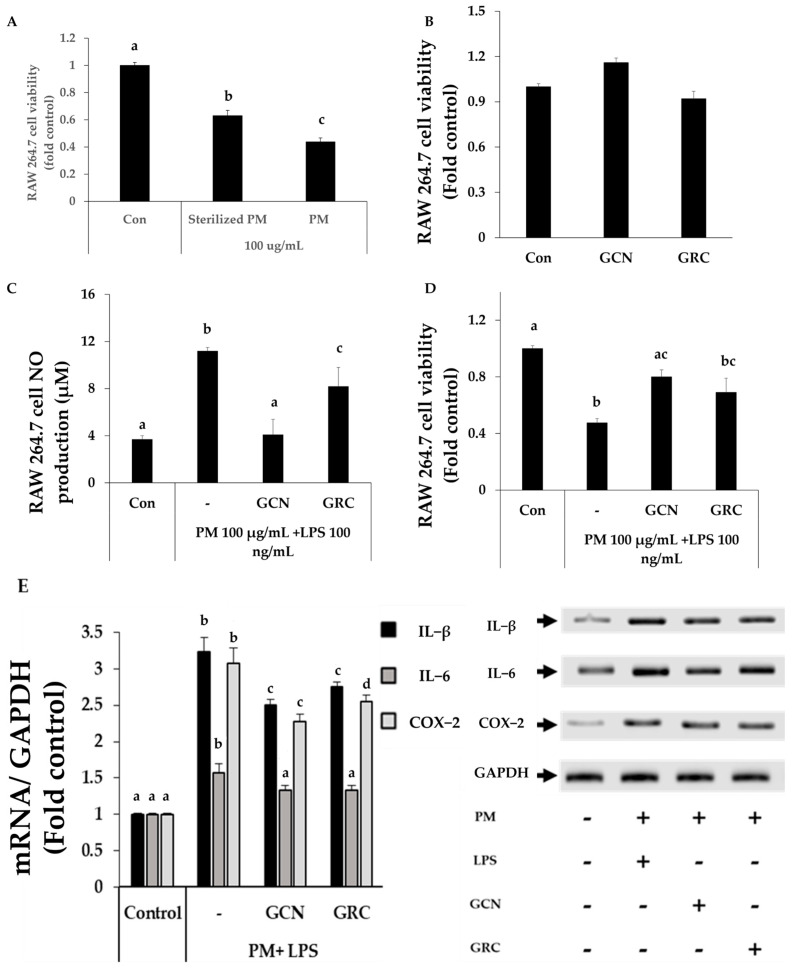
GCN inhibits particulate matter (PM)- and lipopolysaccharide (LPS)-induced RAW264.7 cell inflammatory mediator expression. (**A**) The viability of RAW264.7 cells exposed to wet heat sterilized PM and unsterilized PM (100 μg/mL) was determined by the cell counting kit-8 (CCK-8) assay. (**B**) Viability of RAW264.7 cells after GCN and GRC (200 μg/mL) treatment was measured using a CCK-8 assay. (**C**) Nitric Oxide (NO) production in PM (100 μg/mL)- and LPS (100 ng/mL)-treated RAW 264.7 in the presence or the absence of GRC or GCN (200 μg/mL). (**D**) Cell viability of GRC and GCN (200 μg/mL) treatment in PM (100 μg/mL) and LPS (100 ng/mL) stimulated RAW264.7. (**E**) Effects of GCN and GRC (200 μg/mL) on the levels of inflammatory cytokine mRNAs in RAW 264.7 cells treated with PM (100 μg/mL) and LPS (100 ng/mL). The experiments were repeated three times and results are presented as mean ± SD. ^a–d^ Bars with different letters differ significantly at *p* < 0.05 by Tukey HSD test.

To investigate whether GCN possesses anti-inflammatory effects, we treated GCN on PM-treated RAW264.7 cells. RAW 264.7 cells were exposed to unsterilized PM and sterilized PM (100 μg/mL), respectively. The cell viability of unsterilized PM-treated RAW 264.7 cells was significantly lower than that of the sterilized PM-treated RAW264.7 cells (*p* < 0.05) (Figure 5A). Sterilized PM showed lower cytotoxic effect on RAW 264.7 cells by eradicating microorganisms and their components, including LPS. Therefore, in order to resolve the microorganism contamination issue, we treated LPS on sterilized PM-treated- RAW264.7 cell for further study [56].

Exposure to PM (100 μg/mL) and LPS (100 ng/mL) decreased RAW 264.7 cell viability, compared to the control group (*p* < 0.05). Pretreatment with GCN significantly rescued PM and LPS-induced cell death (Figure 5C,D). In addition, NO production significantly increased in PM- and LPS-treated cells (*p* < 0.05), while it was decreased in GCN-treated cells (*p* < 0.05). The levels of IL-1β, IL-6, and COX-2 mRNA expression were significantly increased in PM- and LPS-treated cells, while they were decreased in GCN-treated cells (*p* < 0.05).

### 2.6. GCN Inhibits PM-Induced Activation of NF-κB/MAPK Signaling Pathways

**Figure 6:**
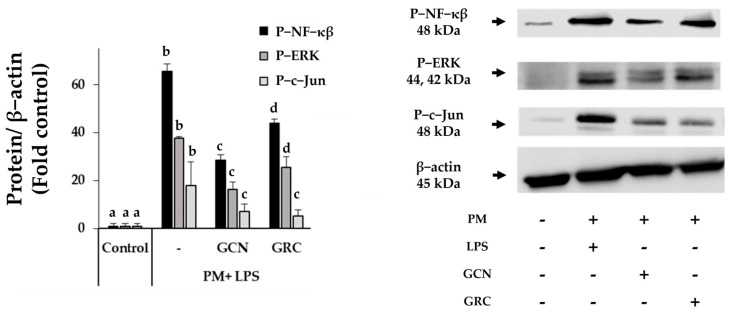
GCN attenuates PM- and LPS-induced inflammatory responses by suppressing the activation of NF-κB and MAPK signaling pathways. RAW 264.7 cells were pre-treated with 200 μg/mL GRC or GCN for 1 h, followed by treatment with PM (100 µg/mL) and LPS (100 ng/mL) for 24 h. Whole-cell lysates were processed for western blot analysis and probed with indicated antibodies. Phosphorylated NF-κB (p-NF-κB), ERK (p-ERK), and c-Jun (p-c-Jun) protein expression levels in RAW 264.7 cells were detected using western blotting. The experiments were repeated three times and results are presented as mean ± SD. ^a–d^ Bars with different letters differ significantly at *p* < 0.05 by Tukey HSD test.

PM exposure activates the NF-κB pathway through TLR and MAPK signals, including JNK and ERK, which are crucial for the regulation of inflammatory gene expression [57,58]. To elucidate the suppressive effect of GCN against PM- and LPS-induced NF-κB/MAPK signaling pathways, we checked the levels of phosphorylated NF-κB (p-NF-κB), ERK (p-ERK), and c-Jun (p-c-Jun) protein expression in RAW 264.7 cells [58,59].RAW 264.7 cells stimulated by PM (100 µg/mL) and LPS (100 ng/mL) showed significant increase in the levels of p-NF-kB, p-ERK, and p-c-Jun protein expressions (*p* < 0.05) (Figure 6). Pretreatment with GCN and GRC (200 µg/mL) significantly reduced the levels of these NF-κB/MAPK signaling proteins. Notably, GCN was more effective than GRC in inhibiting the levels of p-NF-kB, p-ERK, and p-c-Jun protein expressions (*p* < 0.05).

Collectively, these data suggest that GCN can reduce the activation of NF-κB and MAPK signaling pathways in RAW 264.7 cells stimulated by PM and LPS.

### 2.7. Enhanced Bioavailability of GCN Reduces ROS Production in RAW 264.7 Cells

**Figure 7:**
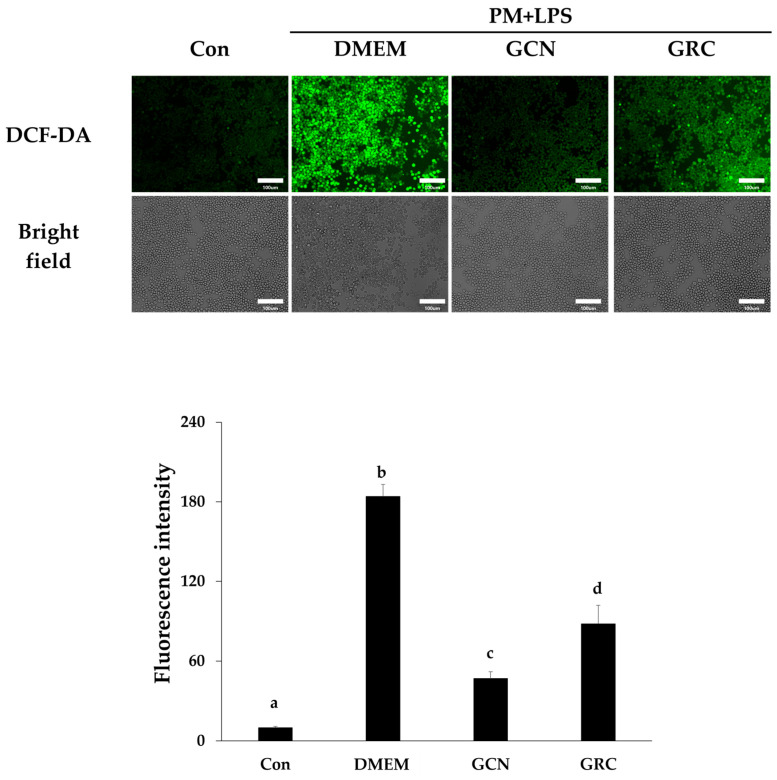
Antioxidant effects of GCN in particulate matter (PM)- and lipopolysaccharide (LPS)-treated RAW264.7 cells. RAW264.7 cells were treated with GRC or GCN (200 µg/mL) for 1 h followed by treatment with PM (100 µg/mL) and LPS (100 ng/mL) for 3 h. Intracellular ROS were detected by fluorescence microscopy (Nikon Eclipse Ti microscope, Point Grey Research, Richmond, BC, Canada) after 2’,7’-dichlorodihydrofluorescein diacetate (DCF-DA) staining, using Metamorph software version 7.8 (Universal Imaging, West Chester, PA, USA; magnification = 200×; scale bar = 100 µm). Relative fluorescence intensities were analyzed using ImageJ software v1.54k. The experiments were repeated three times and results are presented as mean ± SD. ^a–d^ Bars with different letters differ significantly at *p* < 0.05 by Tukey HSD test.

We encapsulated GRC in CN to enhance its bioavailability. Our subsequent investigation focused on determining whether GCN exhibits a significantly improved inhibitory effect on PM- and LPS-induced ROS production compared to GRC alone. As shown in Figure 7, GCN significantly reduced intracellular ROS production compared to PM and LPS treatment (^###^ *p* < 0.001 vs. PM and LPS treated group). Additionally, GCN significantly reduced ROS production compared to GRC (^$$$^ *p* < 0.001 vs. GRC).

**Figure 8:**
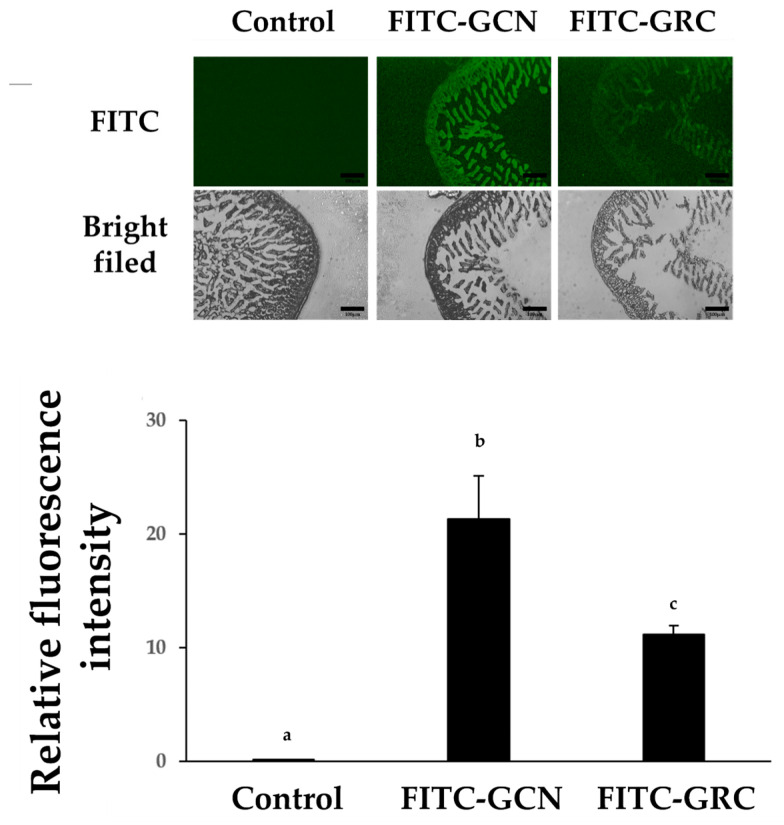
Analysis of GRC encapsulation in chitosan nanoparticles (GCN) mucoadhesion in vivo. Confocal images of excised small intestinal after oral administration of FITC-GRC or FITC-GCN in mice. The dose was equivalent to 300 mg/kg FTIC-GRC or FITC-GCN, and small intestinal sections were taken 3 h after dosage (Scale bar: 100 μm, Magnification: ×100). Relative fluorescence intensities were analyzed using ImageJ software v1.54k. The experiments were repeated three times and results are presented as mean ± SD. ^a–c^ Bars with different letters differ significantly at *p* < 0.05 by Tukey HSD test. PM (particulate matter), GCN (GRC encapsulated in chitosan nanoparticles).

The effectiveness of FITC-GCN as a drug delivery system was evaluated by monitoring intestinal drug absorption in mice. As shown in Figure 8, the fluorescence intensity of FITC-GCN was more intense than that of the FITC-GRC group (^$^ *p* < 0.05 vs. GRC). Additionally, FITC-GCN was detected more intensely in the submucosal and muscle layers, as well as in the mucosal area.

## 3. Discussion

PM refers to a mixture of tiny airborne particles that pose serious health risks, with smaller particles like PM_2.5_ being particularly harmful due to their ability to reach deeper into the lungs compared to larger particles like PM_10_. PM_10_ can enter the respiratory system and reach areas such as the trachea and alveoli, while PM_2.5_ can penetrate even further into lung tissues, leading to significant health risks, including lung and cardiovascular diseases [5,7,8,9,10,60]. PM exacerbates respiratory illnesses, especially in individuals with preexisting conditions like COPD and cystic fibrosis, by triggering inflammation, oxidative stress, and weakening the immune response through biological components, organic compounds, and inorganic compounds such as transition metals [12,13,14,15,16,17,18,20,21,22]. The bioactive compounds in GRC offer a promising strategy for mitigating the detrimental impact of PM_2.5_ on respiratory health. Previously, we demonstrated the efficacy of various bioactive compounds from *R. nulubilis* and *C. militaris* inherent in GRC. Bioactive substances such cordycepin, proteins, soyasaponins, isoflavones, and polysaccharides from GRC have shown potent antioxidant activity, resulting in decreasing PM-induced cell death, demonstrated by their ability to scavenge free radicals [26,31,34,61]. These compounds have also increased the levels and activities of CAT, SOD, and glutathione peroxidase and reduced lipid peroxidation [62,63,64]. However, bioactive ingredients derived from natural products, such as phenolic compounds and cordycepin, often face challenges of low bioavailability due to physicochemical and enzymatic instability and low drug permeability. To resolve this issue, GRC extract was encapsulated in CNs.

We screened the optimal CNs by ionic cross-linking between TPP and CHI. The average diameter and zeta potential of CNs can be adjusted based on the *w/w* ratio of CHI:TPP and the pH of TPP [52,53]. It was demonstrated that a higher CHI ratio results in a larger particle size and standard deviation due to cross-linking between the preformed and newly formed particles, whereas a higher TPP ratio results in a smaller particle size due to the formation of a dense bond with the CHI amino group [53]. In this study, both the size and zeta potential of nanoparticles increased with increasing concentration of CHI (except CN4). Previous studies demonstrated that 100–200 nm size nanoparticles are optimal for cellular uptake [65,66]. Nanoparticles smaller than 200 nm are more efficiently internalized by cells via clathrin-mediated endocytosis, leading to enhanced therapeutic efficacy [66]. Consistent with these findings, the GCN (8 mg/mL) in this study had an average size of 146.1 nm, which would facilitate cellular uptake and improve the bioavailability of GRC components. Furthermore, the zetapotential of CNs showed a positive charge ranging from +2.2 to +22.77 mV, which is attributed to the protonation of the amino groups of CHI and is beneficial for cellular uptake [66]. The zeta potential increased slightly as the pH decreased from 9.8 to 2, suggesting that the reduction in TPP’s negative charge lowered its reactivity with CHI, thereby exposing more amino groups (NH_3_^+^) on the CN surface [67].

In our findings, CNs containing different GRC concentrations showed an increase in negative zeta potential at higher levels. According to prior research, the negative zeta potential of GCNs observed at GRC concentrations (60 and 120 mg/mL) may be due to the migration of negatively charged compounds in GRC such as polysaccharides [68], nucleotides [69], and phenolic compounds [70] to the surface of GCNs [40,68]. A high zeta potential (positive or negative) aids stability by inhibiting agglomeration between particles and is typically optimal within the range of ±20 to 30 mV [54]. Interestingly, the zeta potentials of GCN were +35.6, +34.41, and +30.68 mV at GRC concentrations of 2, 4, and 8 mg/mL, respectively. This result indicates that the stability of the GRC-encapsulated nanoparticles was enhanced compared to empty nanoparticles produced under the chosen CN6 (+22.77 mV) conditions. Notably, GRC 8 mg/mL was used in subsequent analyses because it showed good capture and loading efficiencies of 31.4 ± 1.35% and 7.6 ± 0.33%, respectively. GRC 8 mg/mL demonstrated superior entrapment and loading efficiencies of 31.4 ± 1.35% and 7.6 ± 0.33%, respectively, and was used for the selection subsequent analysis.

PM induces inflammatory cytokines from epithelial tissues, macrophages, neutrophils, and lymphocytes in the respiratory tract, which further attract additional inflammatory cells, exacerbating inflammatory reaction in the pulmonary system, including BALF [55,71,72,73]. Previous studies from our group have demonstrated that GCN includes bioactive compounds such as polysaccharides, cordycepin, and novel isoflavonoids from GRC [26,31,74,75,76]. Among them, cordycepin is reported to prevent airway inflammation and reduce inflammatory cell infiltration in the BALF by decreasing ICAM-1, IL-4, and IL-5 [77]. In addition, we reported that GRC inhibited inflammatory cell infiltration in the inflamed murine skin with type IV hypersensitive reaction [28,64]. In this study, the decreased inflammatory cell population in the BALF and lung tissue histologic observations following GCN administration indicate a significant reduction in PM-induced lung injury. In our in vivo experiments, we used non-sterilized PM to replicate natural environmental exposure conditions. Although this approach closely simulates real-world conditions, it can make it more challenging to pinpoint the specific mechanisms behind PM-induced effects because of the intricate combination of biological and chemical PM components. To address these challenges, in our in vitro studies, we first observed that sterilized PM exhibited reduced cytotoxicity compared to non-sterilized PM. This finding suggests that the biological components present in PM significantly contribute to its overall toxicity. LPS, a component of Gram-negative bacteria that is often found in high concentrations on PM, plays a crucial role in triggering inflammatory responses. Many studies have highlighted the significant contribution of PM containing LPS to the development of inflammatory lung diseases, including COPD [78,79]. To better understand the mechanisms behind PM-induced inflammatory responses, in vitro studies used sterilized PM in combination with LPS to investigate the inflammatory reactions commonly associated with PM exposure.

Exposure to PM damages the first line of defense innate cells, such as macrophages, monocytes, neutrophils, and epithelial cells [80,81]. Primary alveolar macrophages or MH-S cells present several practical challenges, including limited commercial availability, maintenance difficulties, and variability across experiments when studying PM-induced inflammatory diseases [82]. Given the substantial evidence in the literature supporting RAW264.7 cells as a well-established and reliable model for investigating inflammation and oxidative stress in response to PM exposure, we opted to use the commercial RAW264.7 cell line as a representative macrophage model [83,84,85,86,87,88,89].

NO is a key mediator in physiological processes, especially in inflammation, primarily produced by macrophages [90]. Normally, NO aids in blood vessel dilation, platelet aggregation prevention, and apoptosis regulation [91]. However, upon PM exposure, excessive NO production via the inducible nitric oxide synthase (iNOS) pathway drives inflammation, which is particularly significant in respiratory inflammatory diseases like asthma and bronchiectasis, where increased NO leads to harmful reactive nitrogen species (RNS), worsening lung disease progression [92,93,94].

Additionally, it can induce the expression of proinflammatory cytokines such as TNF-α, IL-1β, IL-5, and IFN-γ, all of which were increased in lung tissue after PM exposure in our study [72,95,96,97]. TNF-α and IL-1β, primarily produced by macrophages, further stimulate lung epithelial cells to produce secondary cytokines and cell adhesion molecules [96,98,99]. This cascade leads to the infiltration of inflammatory cells into lung tissue, intensifying the inflammatory response [100,101]. IL-5, mainly produced by T helper cells and mast cells, not only promotes eosinophil activation and recruitment but also activates the JAK/STAT signaling pathway, enhancing the infiltration and differentiation of Th cells [102,103,104,105,106,107]. Our findings suggest that increased inflammatory-cell infiltration in lung tissue and BALF is due to the induction of proinflammatory cytokines from PM-stimulated cells, including macrophages, airway epithelial cells, monocytes, and neutrophils [72,80,106]. Future studies should focus on analyzing in detail how various components of PM induce cytokine production in different immune cells.

Moreover, the NF-κB pathway, activated by IL-1β or TNF-α, triggers the release of additional proinflammatory cytokines including TNF-α, IL-1, and IL-6 [108]. IL-5 also engages in this inflammatory network by activating Lyn, Syk, and JAK2 through IL-5R on eosinophils and boosting the transcription of IL-1β, TNF-α, and IL-5 through the Ras-MAPK and JAK/STAT pathways [104,105,109,110]. This results in the amplification of inflammatory factors in lung tissue [108,109,110,111]. Our previous studies reported that active components of GRC such as polyphenols and isoflavones (daidzein 7-O-β-d-glucoside 4”-O-methylate, glycitein 7-O-β-D-glucoside 4”-O-methylate, genistein 7-O-β-D-glucoside 4”-O-methylate and genistein 4’-O-β-D-glucoside 4”-O-methylate) inhibit T-cell activation by reducing the levels of pro-inflammatory cytokines and chemokines such as IL-6 and IL-1β in mice with type 4 hypersensitivity [64]. In addition, components such as adenosine, cordycepin, and polyphenols in *C. militaris* suppressed the mRNA expression of TNF-α and IL-1β in LPS-stimulated macrophages by downregulating the NF-κB/MAPK pathway [28]. Our data suggest that the reduction of inflammatory cytokine production by GCN reduced lung inflammation that might be mediated through the inhibition of the NF-κB/MAPK inflammatory pathways by GRC components. To further explore these possibilities, we investigated the effect of GCN on PM-stimulated RAW264.7 macrophage cells.

PM can contain LPS, a major component of Gram-negative bacteria, which promotes the production of pro-inflammatory cytokines. LPS is ubiquitous in the environment and can be found in high concentrations on PM, and adherent LPS can induce changes in lung function more than PM alone [78,112]. LPS mediates CD14 and Toll-like receptors to trigger an inflammatory cytokine response in macrophages, which is a major factor in exacerbating inflammatory diseases [113,114,115].

PM can contain high concentrations of LPS, a major component of Gram-negative bacteria, which promotes the production of pro-inflammatory cytokines [101]. LPS is ubiquitous in the environment and can be found in high concentrations on PM, and adherent LPS may induce changes in lung function more than PM alone [100]. LPS mediates CD14 and Toll-like receptors to trigger an inflammatory cytokine response in macrophages, which is a major factor in exacerbating inflammatory diseases [102,103,104].

Therefore, the reduced cytotoxicity in RAW 264.7 cells exposed to sterilized PM in our results might be primarily due to the inactivation of biological components such as Gram-negative bacteria and LPS, which play a crucial role in inducing inflammation during the sterilization process [56]. Thus, in our additional experiments, we co-treated PM and LPS to better understand the pro-inflammatory capabilities of these components.

The rise in inflammatory markers in macrophages following PM exposure can be attributed to both its biological and non-biological components. The biological elements, including Gram-negative bacteria within PM, activate TLR4, leading to enhanced cellular inflammatory responses [116]. Additionally, PM contains high levels of endotoxins that induce significant cytotoxicity and inflammation [115]. Non-biological components such as Fe, Cu, polycyclic aromatic hydrocarbons (PAHs), and quinoline are recognized by various macrophage receptors (e.g., TLR2/4, NLR, and scavenger receptors). This recognition leads to an increased expression of TNF-α, IL-1β, IL-6, and COX-2 [117,118]. The pro-inflammatory cytokines IL-1β and IL-6, produced by macrophages, recruit and activate leukocytes, promoting an inflammatory response [96,119]. Additionally, the enzyme COX-2 promotes vasodilation and increases vascular permeability, which facilitates the infiltration of inflammatory cells [120]. Our study indicates that GCN pretreatment notably decreased IL-1β, IL-6, and COX-2 levels, elevated by PM and LPS exposure, suggesting a reduction in inflammatory cell infiltration in lung tissue and BALF by inhibiting macrophage activation.

Exposure to PM and LPS is crucial for inducing acute inflammatory responses in pulmonary macrophages through the activation of the NF-κB via TLR and MAPK (mainly JNK and ERK) pathways, which regulate the expression of inflammatory genes [57,58]. This mechanism plays a significant role in the onset of lung diseases [121]. PM and LPS also cause oxidative stress by producing ROS, such as superoxide anions, nitric oxide, and hydrogen peroxide [23,122]. ROS generated by neutrophils, macrophages, and structural cells (epithelial and endothelial cells) involved in PM-induced inflammatory processes disrupt the balance between the antioxidant enzymes CAT and SOD and promote inflammation by activating the redox-sensitive MAPK and NF-κB pathways [20,23,123].

We investigated whether GCN reduces ROS and NF-κB related signaling molecules involved in PM-induced inflammation. Our findings indicate that GCN pretreatment significantly blocked the activity of MAPK (JNK and ERK) and NF-κB pathways in PM- and LPS-exposed macrophages, reducing the production of proinflammatory cytokines. This suggests that GCN has therapeutic potential in the management of PM-related inflammatory lung disease.

LPS, which is often found at significant levels in non-sterile PM, is known to induce ROS production in macrophages, produce inflammatory cytokines in leukocytes, and induces ROS production in endothelial cells through NAD(P)H oxidase (Nox) activation [112,124,125]. We observed a significant increase in intracellular ROS levels in macrophages exposed to PM and LPS. Additionally, SOD-1 protein expression levels were significantly reduced in PM-exposed mouse lungs.

The increase in ROS in macrophages exposed to PM may be associated with the increase in inflammatory cytokines such as IL-1β and IL-6 by activating NF-κB and MAPK pathways [20,123]. These results are consistent with previous studies showing that PM and LPS can induce oxidative stress and inflammation through similar mechanisms [23,124,125]. These pathways contribute to cellular apoptosis through both extrinsic and intrinsic mechanisms, leading to lung toxicity, alveolar wall damage, emphysema, and COPD [16,67,68,71]. Previously, we reported that GRC contains a variety of bioactive substances derived from *C. militaris* and *R.* nulubilis [25,26,27,28,126,127]. GRC contains various bioactive compounds, including cordycepin, polysaccharides, polyphenols such as proanthocyanidins, and recently identified isoflavones (genistein 4-O-β-D-glucoside 4”-O-methylate, daidzein 7-O-β-D-glucoside 4”-O-methylate, genistein 7-O-β-D-glucoside 4”-O-methylate, and glycitein 7-O-β-D-glucoside 4”-O-methylate), all recognized for their potential to reduce ROS levels [31,34,35,36]. Polysaccharides from *C. militaris* demonstrate significant scavenging activity against hydroxyl and superoxide radicals, playing a key role in directly scavenging ROS [26]. Additionally, phenolic compounds from *C. militaris* can chelate intracellular iron, thereby preventing ROS formation through the Haber-Weiss/Fenton reactions [26,128]. Isoflavones in *R. nulubilis* inhibit lipid peroxidation, scavenge free radicals, and chelate metal ions, thereby enhancing antioxidant capacity [129]. Components of GRC also affect cell signaling pathways associated with oxidative stress and inflammation. Phenolic compounds interfere with oxidative stress signaling and inhibit the NF-κB and MAPK pathways, thus reducing inflammatory responses [33]. Cordycepin, the main active component of *C. militaris*, has been demonstrated to enhance the activity of antioxidant enzymes such as SOD and glutathione peroxidase (GSH-Px) in PC12 cells treated with 6-OHDA, providing protection against oxidative damage [32]. Additionally, cordycepin has been shown to inhibit the phosphorylation of ERK1 at Thr^202^ and Tyr^204^ and reduce nuclear levels of NF-κB, further contributing to its anti-inflammatory effects [130]. This inhibition can potentially reduce COX2 production by blocking the TLR4/NF-κB signaling pathways, which may lead to decreased levels of cytokines IL-1β and TNF-α [131]. Our findings support this, showing a significant reduction in ROS levels in macrophages exposed to PM and LPS following treatment with GRC and GCN. Additionally, this reduction was associated with lower levels of inflammatory markers such as IL-1β and COX2, as well as decreased activity in the NF-κB and MAPK signaling pathways.

In this study, we observed that the fluorescence signal in the intestinal tissue of mice given the FITC-GRC solution orally was significantly lower than that in mice given FITC-GCN. Several factors may contribute to the low bioavailability of FITC-GRC, which could potentially be improved by encapsulating it in chitosan nanoparticles.

First, many bioactive compounds in GRC, such as flavonoids and polyphenols, exhibit poor water and lipid solubility, leading to low absorption rates in the gastrointestinal tract and poor permeability across lipid-rich biological membranes [132,133]. Chitosan nanocarriers exhibits ideal properties for drug delivery systems, especially for poorly soluble drugs. Therefore, FITC-GCN may have improved the solubility of GRC components as an efficient carrier to enhance the bioavailability of hydrophobic and labile active compounds [134,135].

Second, the absorption of water-soluble components, such as phenolic compounds, faces considerable obstacles in traversing the intestinal lipid membrane. This is primarily attributed to the hydrophilic nature of the stagnant aqueous layer covering the intestinal mucosa, serving as a significant barrier. Drug molecules larger than approximately 0.4 nm also face challenges passing through these aqueous channels [136,137].

The permeability of water-soluble components was improved by CN. The positively charged CNs interact strongly with the negatively charged cell membranes, which improves the transport of polar drugs across epithelial surfaces [135,138,139]. Additionally, CNs facilitate intestinal penetration by opening tight junctions between cells and improving mucosal adhesion and penetration [140]. Therefore, CNs enhance the permeability of water-soluble components in GRC by promoting intestinal permeation through these mechanisms.

Third, the large molecular size of some plant compounds, such as flavonoids, and glycoside aglycones, reduces oral absorption since they are too large to be absorbed via passive diffusion [133,141]. Specifically, flavonoids are mostly found in glycoside forms with a high molecular weight, leading to poor absorption. These compounds are hydrolyzed in the intestine to less absorbable forms, further reducing their bioavailability [132]. This could be enhanced by the reduced size of GCN. The nanometer scale of GCN enables active compounds of GRC to traverse biological barriers more effectively, enhancing drug targeting and efficacy [142].

Finally, cordycepin and adenosine, the main bioactive compounds in GRC, are rapidly metabolized by adenosine deaminase, resulting in the production of inactive metabolites [50]. CNs increase the stability of the drug by preventing enzymatic degradation and maintaining controlled and sustained drug release [143]. The chemical bonding and structural integrity of GCN are pH-sensitive, allowing for controlled release of GRC components [65,138,139]. Previous research has demonstrated that CNs elevate the bioavailability of bioactive substances, enhancing their ability to scavenge ROS [144,145].

## 4. Materials and Methods

### 4.1. Synthesis of Chitosan Nanoparticles (CN) and GRC Chitosan Nanoparticles (GCN)

CNs were prepared using the ionotropic gelation method with sodium triphosphate pentabasic (TPP) as described by Masarudin et al. [67,146]. A chitosan (CHI) solution was prepared by dissolving 200 mg of CHI powder in 50 mL of deionized distilled water (4 mg/mL). Acetic acid (0.5 mL) was added dropwise over 1 h under magnetic stirring at 600 rpm to completely dissolve the powder. The homogenized CHI solution at concentrations of 1 and 4 mg/mL was adjusted to pH 5.5 with 0.5 M NaOH and filtered through a 0.45-μm vacuum filter. To prepare the TPP solution, sodium triphosphate was dissolved in deionized distilled water at a concentration of 1 mg/mL and adjusted to pH 2 and 9.8 using 1 M HCl or 0.1 M NaOH. The prepared TPP solution was filtered through a 0.45-μm syringe filter. CN was synthesized at various CHI:TPP *w/w* ratios (1:1, 2:1, and 4:1).

For CN synthesis, the pH of distilled water was adjusted to 5.5. Briefly, 6 mL of distilled water was added to the CHI solution and stirred at 600 rpm for 5 min. Then, 6 mL of pH 2 or 9.8 TPP solution was added dropwise to the stirred CHI solution at 1 mL/min for 6 min using a syringe pump (NE-300; Just Infusion Syringe Pump; New Era Pump Systems Inc., Farmingdale, NY, USA), followed by stirring for 30 min. To obtain CNs, the solution was centrifuged at 15,000 rpm for 30 min, and the obtained pellet was completely dissolved in 5 mL of 10% sucrose solution for lyophilization. The supernatant was used to calibrate the amount of unencapsulated GRC in CNs for further experiments.

GCN was synthesized by adding various concentrations of GRC extract (2, 4, 8, 15, 30, 60, and 120 mg/mL) instead of distilled water under optimal CN synthesis conditions (TPP mass ratio = 4:1, TPP pH 2, CN6 condition). Consequently, the synthesized GCNs were collected from solution by centrifugation at 15,000 rpm for 30 min, freeze-dried, and used in powder form for subsequent experiments.

### 4.2. NTA

Particle sizes and zeta potentials of CN and GCN were measured using a ZetaView PMX120 instrument (Particle Matrix GmbH; Inning am Ammersee, Bavaria, Germany) at the Bionano Materials Research Center of Gachon University, as previously described [147]. The samples were diluted in distilled water and analyzed at room temperature using the ZetaView Particle Matrix software version 8.05.12 SP2. For particle size measurements, we used the scatter mode with a 488 nm laser, conducting three cycles at 11 frames per cycle with a sensitivity of 90, shutter value of 100, and video frame rate of 30. The zeta potential was analyzed over five cycles at two frames per cycle, using a sensitivity of 90, shutter value of 100, and video frame rate of 60. Each measurement consisted of approximately 2000–2500 tracks. Prior to analysis, the pre-measured contents were removed, and the samples were injected into the sample chamber using a sterile syringe.

### 4.3. PM Sample and Cell Culture Preparation

PM was collected based on the method described in the previous study [31]. To obtain PM_2.5_ particles for in vitro and in vivo experiments, HEPA filters that captured PM_2.5_ were placed in a 50 mL tube with 10 mL of 75% alcohol. The mixture was then sonicated for 30 min at 4 °C using a sonicator (powersonic 610, KLEENTEK, Maroochydore, Queensland, Australia). The sonicated sample was then filtered to isolate particulate matter smaller than 2.5 μm (PM_2.5_) using filter paper (1001-110, Whatman, Maidstone, Kent, UK). The filtered suspension was concentrated with a reduced pressure concentrator and stored at −80 °C until further use.

The RAW 264.7 cells were purchased from the Korean Cell Line Bank (KCLB, Seoul, Republic of Korea). Cells were maintained in Dulbecco’s modified Eagle’s medium (DMEM, Welgene, Daegu, Republic of Korea) supplemented with 10% fetal bovine serum (Welgene) and 1% penicillin and streptomycin (Welgene). They were cultured in a 75 cm^2^ cell culture flask at 37.5 °C in humidified 5% CO_2_ incubators.

### 4.4. In Vivo PM Exposure

**Figure 9:**
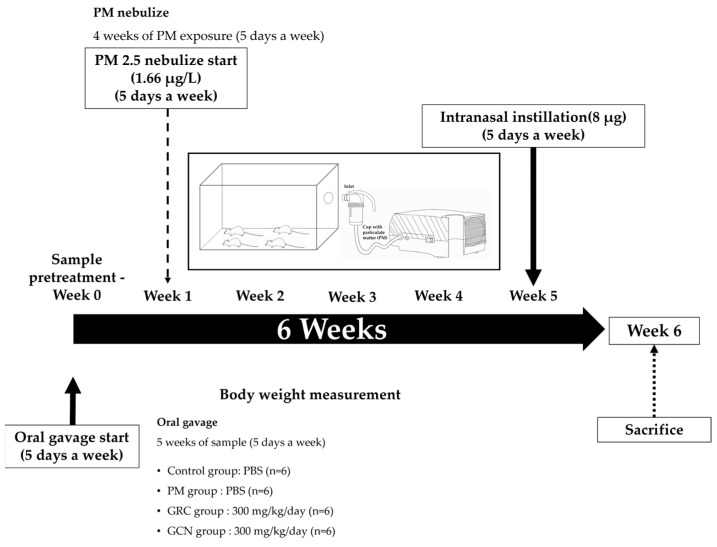
Particulate matter (PM) nebulize schedule. Mice were exposed to PM via inhalation for 4 weeks, followed by an additional week of intranasal instillation (5 days/week, *n* = 6 per group). Mice were treated by oral gavage with 100 μL of PBS or sample (300 mg/kg) daily for 6 weeks (5 days/week) and tissues were collected 2 days later for analysis.

Animal experiments were conducted in accordance with the guidelines of the Institutional Animal Care and Use Committee (IACUC) of Gachon University (Gyeonggi, South Korea), under approval number GU1-2022-IA0023-01. Female BALB/c mice aged six weeks were purchased from JA BIO (Suwon, Gyeonggi, South Korea) and acclimatized for one week at 20 ± 2 °C before the experiment, with free access to standard laboratory chow and water under a 12:12 h light–dark cycle (lights on at 06:00). After an acclimation period, referring to methods like those of Vignal et al., we divided the mice into four groups (*n* = 6). Over the course of four weeks (five days per week), we exposed them to PM using the PARI BOY^®^ SX nebulizer (PARI GmbH, Starnberg, Germany). A PM solution with a concentration of 132.8 µg/mL was nebulized at a rate of 0.15 ml/min into an 11 L chamber for 40 min each day, resulting in a total PM content of 796.8 µg per day and an exposure concentration of 1.66 µg/L, or exposed the control group to distilled water [148]. Additionally, PM (8 μg in 40 μL PBS) was administered intranasally for an additional two weeks. The average PM level in Seoul is 27 μg/m^3^, equivalent to a daily human exposure amount of 287 μg [149,150]. Based on respiratory volume, to mimic the air pollution levels of Seoul, mice would need to be exposed to approximately 8 μg per day [150] (Figure 9).

For six weeks (five days per week), mice were orally administered 100 μL PBS or samples of GCN and GRC (300 mg/kg) daily. At the end point, animals were anesthetized with Avertin (intraperitoneally at 100 mg/kg) and euthanized via cardiac puncture. Lungs were perfused with PBS to obtain bronchoalveolar lavage fluid (BALF). Subsequently, the lungs were harvested and stored at −80 °C for RT-PCR and western blot analyses or fixed in 10% formaldehyde for histological evaluation and then embedded in paraffin blocks.

### 4.5. Immunohistochemistry

Immunohistochemical analysis was conducted as previously described [64], using primary antibodies against CD4 (1:100 dilution, Cell Signaling Technology Inc., Danvers, MA, USA) and F4/80 (1:50 dilution, Invitrogen, Carlsbad, CA, USA) for 1 h at 25 °C. Following primary antibody incubation, sections were washed and then incubated with a biotinylated anti-rabbit secondary antibody (DAKO, Camarillo, CA, USA) for 30 min. A horseradish peroxidase (HRP)-streptavidin complex was subsequently applied to detect the secondary antibody for another 30 min. Chromogenic detection was conducted using the 3,3’-Diaminobenzidine (DAB) chromogen kit (Vector Laboratories, Burlingame, CA, USA), and sections were counterstained with 1% methyl green for 1 min. Slides were examined under a Nikon Eclipse Ti microscope equipped with a color digital camera (Point Grey Research, Richmond, BC, Canada) and images were analyzed with MetaMorph software version 7.8 (Molecular Devices, Sunnyvale, CA, USA).

### 4.6. Hematoxylin and Eosin (H&E) Stain

H&E analysis was performed as previously described [151]. Briefly, formalin-fixed lung tissues embedded in paraffin blocks were sectioned into 5 μm slices. Each paraffin-embedded tissue section underwent deparaffinization using Xylene (Junsei Chemical Co., Ltd., Tokyo, Japan), followed by progressive rehydration through a graded ethanol series of 100%, 95%, and 70%. The nuclei were stained for 4 min using Hematoxylin solution (TissuePro Technology, Gainesville, FL, USA). The stained tissues were differentiated in 0.3% acid alcohol and then rinsed under running tap water. Subsequently, staining was performed with Eosin solution (TissuePro Technology) for 2 min, followed by a dehydration process through a graded series of ethanol at 70%, 95%, 100%, and finally Xylene. After drying in a fume hood, histopathological alterations in the tissues were observed under a light microscope. Digital microscopic images were captured at a fixed magnification of 200×, focusing on representative areas.

### 4.7. Bronchoalveolar Lavage Fluid (BALF) Analysis

The bronchoalveolar lavage fluid (BALF) was centrifuged at 1500 rpm for 5 min to separate cells. For the evaluation of BALF cells, 1 mL of erythrocyte lysis buffer was added to the isolated cells, followed by centrifugation at 1500 rpm for 5 min. The cells were then resuspended in 500 µL of RPMI and aliquoted onto slides at a concentration of 0.5–1.0 × 10^5^ cells per 300 µL. After drying for 2 h, cells were stained using a Diff-Quick kit (Thermo Fisher Scientific, Waltham, MA, USA) and observed under a 100x magnification using an eclipse Ti-S inverted microscope (Nikon, Tokyo, Japan) for the quantification of macrophage, neutrophils, and total cell counts. Images were captured using Metamorph software version 7.8 (Universal Imaging, West Chester, PA, USA). The images presented are representative of three independent experiments.

### 4.8. Cell Viability Assay

RAW264.7 cells were evaluated using the cell counting kit-8 (CCK-8) assay (Dojindo Laboratories, Kumamoto, Japan), as described previously [31,152]. Cells (2 × 10^4^ cells/well) were seeded in 96-well plates. RAW264.7 cells were treated in the presence or absence of sterilized PM (heated at 121 °C for 20 min with humidification) and non-sterilized PM, each at a concentration of 200 µg/mL, and then incubated for 24 h. In addition, RAW264.7 cells were pre-treated with GCN and GRC, each at 200 µg/mL, for 1 h before treating sterilized PM (200 µg/mL) and LPS (100 ng/mL) After 24 h incubation, 10 µL of CCK-8 solution was added to each well, and then allowed to react with RAW 264.7 cells for 1 h. The optical density (OD) was measured using a microplate reader at a wavelength of 450 nm.

### 4.9. Measurement of Nitric Oxide (NO) Concentration

Released amount of NO from RAW 264.7 macrophages was measured using the Griess reaction assay as described previously [28]. RAW 264.7 macrophages (2 × 10^4^ cells/well) were treated with 200 μg/mL of GRC or GCN for 1 h and then treated with PM and LPS for 24 h. Next, 50 μL of the medium was mixed with Griess reagent (1% sulfanilamide, 0.1% naphthylenediamine dihydrochloride, and 0.5% H_3_PO_4_). The optical density was measured using a microplate reader (Epoch, Biotek Instruments, Inc., Winooski, VT, USA) at 450 nm.

### 4.10. RNA Isolation and RT-PCR

Total RNA was isolated from tissue samples and RAW 264.7 cells treated with PM (100 μg/mL) and LPS (100 ng/mL) as previously described [152]. The PCR protocol was initiated with an initial denaturation step at 94 °C for 2 min, followed by a cycling protocol of 30 cycles, which included denaturation at 94 °C for 30 s, annealing at 55 °C for 30 s, and extension at 68 °C for 1 min. A diverse array of primers was utilized for amplifying specific gene targets as follows: GAPDH (forward: 5’ GAA GGT CGG TGT GAA CGG AT 3’, reverse: 5’ ACT GTG CCG TTG AAT TTG CC 3’), IL-1β (forward: 5’ GAG TGT GGA TCC CAA GCA AT 3’, reverse: 5’ CTC AGT GCA GGC TAT GAC CA 3’), IL-5 (forward: 5’ TCT GAT CCT CCT GCC TCC TC 3’, reverse: 5’ GAT CCT CCT GCG TCC ATC TG 3’), IL-6 (forward: 5’ TCC AGT TGC CTT CTT GGG AC 3’, reverse: 5’ AGC CTC CGA CTT GTG AAG TG 3’), TNF-α (forward: 5’ AAG CCT GTA GCC CAT GTT GTA G 3’, reverse: 5’GAT GGC AGA GAG GAG GTT GAC 3’), INF-γ (forward: 5’ CA CAC TGC ATC TTG GCT TTG 3’, reverse: 5’ TC CAC ATC TAT GCC ACT TGA G 3’), COX-2 (forward: 5’ ATT ACT GCT GAA GCC CAC CC 3’, reverse: 5’ GGC CCT GGT GTA GTA GGA GA 3’) and iNOS (forward: 5’ CTT CAA CAC CAA GGT TGT CTG CA 3’, reverse: 5’ ATG TCA TGA GCA AAG GCG CAG AA 3’). The levels of these expressed genes were normalized to the housekeeping gene GAPDH to ensure accuracy and reliability in the quantification of gene expression changes.

### 4.11. Western Blot Analysis

Western blotting was conducted in accordance with previously described methods [31,153]. In brief, RAW 264.7 cells were lysed in radioimmunoprecipitation assay (RIPA) buffer (Cell Signaling, MA, USA) and subsequently homogenized. Proteins were isolated by centrifugation at 14,000× *g* for 10 min.

Protein concentrations were quantified using the Pierce bicinchoninic acid (BCA) protein assay kit (Thermo Fisher Scientific, Waltham, MA, USA). Equal amounts of proteins were resolved by 10% sodium dodecyl sulfate-polyacrylamide gel electrophoresis (SDS-PAGE). The separated proteins were then transferred onto nitrocellulose membranes (Bio-Rad Laboratories, Inc., Hercules, CA, USA) and blocked with 5% bovine serum albumin (BSA) for 1 h at room temperature. Overnight incubation at 4 °C was done with the membranes immersed in Tris-buffered saline with Tween (TBS-T, 20 mM Tris, 500 mM sodium chloride, pH 7.6, 0.1% Tween 20) containing primary antibodies against phosphorylated (p)-ERK (1:1000; Cell Signaling, Danvers, MA, USA), phosphorylated (p)-c-Jun (1:1000; Cell Signaling, Danvers, MA, USA), phospho-NF-κB (1:1000; Cell Signaling, Danvers, MA, USA), and beta-actin (1:1000; Cell Signaling, Danvers, MA, USA) diluted as per the protocols. This was followed by a 1 h incubation with horseradish peroxidase (HRP)-linked anti-rabbit IgG secondary antibody (1:2000; Cell Signaling, Danvers, MA, USA). The blots were visualized using an enhanced chemiluminescence detection solution (EzWestLumi plus, ATTO corporation, Tokyo, Japan) and images were captured and analyzed using Odyssey LCI Image software (LI-COR Biosciences, Lincoln, NE, USA). The blots shown are representative of at least three repeats.

### 4.12. Reactive Oxygen Species (ROS) Assay

The generation of reactive oxygen species (ROS) was measured using the method described previously [1]. Briefly, intracellular ROS levels were measured with 25 μM DCFH-DA, which forms fluorescent DCF upon reacting with ROS, following the manufacturer’s protocol (ab113851, Abcam, Cambridge, UK). After treating cells in 6-well plates with DCFH-DA and incubating at 37 °C for 45 min, fluorescence was detected at 100× using a Nikon Eclipse Ti-S microscope and Metamorph software version 7.8, with ImageJ software v1.54k quantifying the fluorescence intensity to indicate ROS levels.

### 4.13. Ex Vivo Absorption Study in Mouse

Mouse small intestinal absorption was assessed using previously described methods [154]. Prior to the experiment, mice were fasted for 12 h. They were then orally administered FITC-GRC, FITC-GCN, or FITC aqueous suspension at a dose of 300 mg/kg. FITC-GRC was prepared by adding FITC solution (0.05 mg/mL) to GRC extract (8 mg/mL), followed by centrifugation and resuspension until no fluorescence was detected in the supernatant. FITC-GCN was prepared by adding FITC-GRC (8 mg/mL) to CHI (4 mg/mL) and TPP (1 mg/mL), followed by repeated centrifugation until no fluorescence was detected in the supernatant. Next, 3 h after administration, the mice were sacrificed, and the intestine were immediately excised by laparotomy. The intestines were washed with PBS to clear out any luminal contents, and the tissues were fixed in 4% paraformaldehyde for 1 day before being placed in a 30% sucrose solution until embedding. Subsequently, the small intestine tissues were cryosectioned and examined at 100× magnification using a confocal microscope (Nikon Eclipse Ti-S, Nikon, Tokyo, Japan). The visualization of FITC-GCN and GRC was performed using MetaMorph software version 7.8(Universal Imaging, West Chester, PA, USA).

### 4.14. Statistical Analysis

Data were collected from at least three separate experiments and presented as mean ± standard deviation. Statistical evaluation was performed using one-way analysis of variance followed by Tukey HSD test. Data analysis was performed using SPSS version 12 software (IBM Corp., Chicago, IL, USA).

## 5. Conclusions

This study demonstrated the effectiveness of GCN in reducing lung inflammation and damage caused by PM exposure. PM harms lung tissue and impairs the immune defense system, leading to oxidative stress and inflammatory reactions that worsen a range of respiratory diseases. GCN inhibits the production of PM- and LPS-induced inflammatory cytokines and mitigates inflammation by blocking the NF-κB and MAPK signaling pathways. Additionally, GCN alleviates oxidative stress through increased antioxidant enzyme activity and reduced ROS levels. The primary bioactive components of GRC, such as cordycepin, polysaccharides, polyphenols, and isoflavonoids, demonstrate strong antioxidant and anti-inflammatory properties. These compounds help decrease lung cell death and prevent the infiltration of inflammatory cells into the lungs due to PM exposure. CNs enhance the bioavailability of GRC constituents, facilitating intestinal absorption and cellular uptake. Notably, nano-encapsulation of GRC enhances its permeability through biological membranes, protects it from enzymatic degradation, and allows for sustained drug release. Therefore, this study supports the potential therapeutic utility of GCN in treating PM-related inflammatory lung diseases, calling for further research to elucidate the mechanism of action of GCN more clearly.

## Data Availability

Data are contained within the article.
